# The effects of tumor size and postoperative radiotherapy for patients with adult low‐grade (WHO grade II) infiltrative supratentorial astrocytoma/oligodendroglioma: A population‐based and propensity score matched study

**DOI:** 10.1002/cam4.1853

**Published:** 2018-10-30

**Authors:** Dong‐Dong Lin, Xiang‐Yang Deng, Dong‐Dong Zheng, Cheng‐Hui Gu, Li‐Sheng Yu, Shang‐Yu Xu, Dan‐Dong Li, Jun‐Hao Fang, Bo Yin, Han‐Song Sheng, Jian Lin, Xiao‐Lei Zhang, Nu Zhang

**Affiliations:** ^1^ Department of Neurosurgery The Second Affiliated Hospital and Yuying Children's Hospital of Wenzhou Medical University Wenzhou China; ^2^ Department of Orthopaedics The Second Affiliated Hospital and Yuying Children's Hospital of Wenzhou Medical University Wenzhou China

**Keywords:** astrocytoma/Oligodendroglioma, low‐grade (WHO grade II), postoperative radiotherapy, propensity score match, Surveillance, Epidemiology, and End Results, tumor size

## Abstract

**Background:**

The update of 2018 NCCN guidelines (central nervous system cancers) recommended the risk classification of postoperative patients diagnosed as adult low‐grade (WHO grade II) infiltrative supratentorial astrocytoma/oligodendroglioma (ALISA/O) should take tumor size into consideration. Moreover, the guidelines removed postoperative radiotherapy (PORT) for low risk patients. Our study aimed to explore the specific tumor size to divide postoperative patients into relatively low‐ or high risk subgroups and the effect of PORT for ALISA/O patients.

**Methods:**

We conducted a retrospective study choosing 1277 postoperative ALISA/O patients from the Surveillance, Epidemiology, and End Results database. The X‐tile analysis provided the optimal cutoff point based on tumor size. The differences between surgery alone and surgery +RT groups were balanced by propensity score‐matched analysis. The multivariable analysis and the nomogram evaluated multiple prognostic factors based on cancer‐specific survival (CSS) and overall survival (OS).

**Results:**

X‐tile plots defined 59 mm (*P* < 0.001) as the optimal cutoff tumor size value in terms of CSS, which was verified in multivariate analysis (*P* < 0.001). The Kaplan‐Meier analysis showed that the surgery alone had higher CSS and OS than surgery +RT, while the low risk group had no statistical significance after propensity score match. Multivariable analysis showed that surgery +RT was independently associated with diminished OS and CSS for high risk group, which had no statistical significance for low‐risk group.

**Conclusions:**

Our study suggested that tumor size of 59 mm was an optimal cutoff point to divide postoperative patients into relatively low‐ or high risk subgroups. PORT may not benefit patients, while the effects of PORT for low risk patients need further research.

## 1 INTRODUCTION

WHO grade II astrocytoma and oligodendroglioma are the main components of low‐grade glioma, which accounts for <25% of gliomas and <10% of all CNS tumors.^1^ Although low‐grade astrocytoma and oligodendroglioma are slow‐growing and have a relatively good prognosis, they eventually turn malignant, leading to very different outcomes. Extensive resection is still a preferred treatment. With the development and popularization of magnetic resonance imaging (MRI), it had been the gold standard for brain tumor, which provides a reasonably good delineation of tumors, as this might decide the procedure of surgery or an effective treatment. According to the 2017 NCCN guidelines (CNS cancers),^2^ surgeons should evaluate whether maximal safe resection of a tumor is feasible by MRI for ALISA/O patients.^3^ When maximal safe resection of a tumor is feasible, patients would receive attempted gross total resection, and if not, patients would receive subtotal resection or biopsy. After operation, patients would be divided into low‐ (age ≤40 years and gross total resection) or high risk (age >40 years or no gross total resection) group to select the next treatment.

Recently, the 2018 NCCN guidelines^4^ for ALISA/O redefined high risk standard, recommended to take tumor size into consideration. Some studies revealed tumor size >6 cm was associated with unfavorable prognosis for survival,^5^ while others suggested risk grading of tumor size should refer to primary tumor classification (T classification: tumor size ≤3; 3‐5; >5 cm).^6^, ^7^, ^8^ There were also several artificial classification methods of tumor size.^9^, ^10^ The demarcation of exact tumor size was disputed. What is more, the guidelines removed postoperative radiotherapy (PORT) for low risk patients. The European Organization for Research and Treatment of Cancer (EORTC 22844) conducted a randomized trial, which had shown no difference in CSS and OS for low‐grade glioma patients who received PORT.^6^ Another trial from EORTC 22845 found that PORT improved CSS, but did not affect OS.^11^ The role of PORT for ALISA/O patients remained controversial.

In our study, we used data from the Surveillance, Epidemiology, and End Results (SEER) database^12^ to explore criteria for tumor size to differentiate low‐ and high risk subsets. Meanwhile, we focused on the effects of PORT for ALISA/O patients in low‐ and high risk groups.

## 2 MATERIALS AND METHODS

### 2.1 Data source

Data were obtained from the SEER 18 registries research database, which covers approximately 26% of the total US population from 18 areas of United States.^12^ SEER is committed to reduce the cancer burden among the US population by providing detailed information on cancer statistics. It is supported by the Surveillance Research Program in NCI's Division of Cancer Control and Population Sciences (DCCPS). However, a signed SEER Research Data Agreement form was required to gain access the SEER database. So we submitted a request for access to the database. Once the agreement was accepted, we could download the SEER*Stat software and data files from the SEER database directly.

### 2.2 Cohort selection

When meeting following inclusion criteria, patients were selected in our study: pathological types of astrocytoma (including Histologic Type ICD‐O‐3: “9400: Diffuse astrocytoma,” “9410: Protoplasmic astrocytoma,” “9411: Gemistocytic astrocytoma,” “9420: Fibrillary astrocytoma,” “9424: Pleomorphic xanthoastrocytoma”) or oligodendroglioma (Histologic Type ICD‐O‐3:9500) between January 1988 and December 2013; older than or equal to 18 years old; WHO grade II; history of gross total resection (GTR), subtotal resection (STR), or biopsy; and only one primary tumor. And the exclusive criteria were as follows: receipt of radiation preoperation, unawareness of radiation status, infratentorial tumor, no surgery, or surgical information unknown.

The data from the SEER database contained the baseline demographics of patients (age, sex, race/ethnicity, and year of diagnosis), characteristics of tumors (size, site, and histologic type), and treatment details (surgical type and PORT). In this study, histologic subtypes were classified as astrocytoma and oligodendroglioma. Patients were divided into frontal lobe, temporal lobe, parietal lobe, occipital lobe, and other sites (cerebrum NOS, overlapping lesion, and ventricle) according to the tumor site.

The end points for this study included cancer‐specific survival (CSS) and overall survival (OS) according to specific codes provided by SEER. End points were obtained through 31 December 2013. CSS was defined as the time in months from diagnosis until death as a result of cancer, and OS was defined as the interval from diagnosis until death as a result of any cause.

### 2.3 Statistical analysis

The X‐tile^13^ program was used to determine the tumor size cutoff points, and it identified the cutoff value according to the minimum *P* values from log‐rank chi‐square statistics for the continuous tumor size in terms of CSS. Survival curves were generated using Kaplan‐Meier analysis, and their differences were evaluated by log‐rank test.

Pearson chi‐square test was used to analyze categorical variables, and two‐sample t test was used to analyze continuous variables. Significant differences in the characteristics were balanced by propensity score match (PSM)^14^ (ratio 1:1). The survival curves of CSS and OS were compared by log‐rank test in different groups with the Kaplan‐Meier method. In order to adjust for baseline variables in the comparison, we used a Cox proportional hazards model that included all predictors from the SEER database.

A nomogram based on variables with significant differences in multivariate Cox analysis used the package of rms^15^ in R version 3.5.0. We used the concordance index (C‐index) and compared nomogram‐predicted vs observed Kaplan‐Meier estimates of survival probability to measure and assess the performance of the nomogram. The C‐index was positively correlated with accuracy of the prognostic prediction. During the internal validation of the nomogram, we calculated the total points of each patient based on established nomogram, which were used as a factor by Cox regression in this cohort, and finally, the C‐index and calibration curve were obtained based on the regression analysis.

A two‐sided *P* value of 0.05 was considered statistically significant. Most analyses were executed with SPSS 25.0 (SPSS, Chicago, IL), and the K‐M curve was depicted by GraphPad Prism 6.0 (GraphPad Software, San Diego, CA).

## 3 RESULTS

### 3.1 ***Baseline characteristics of study cohort***

A total of 1277 ALISA/O patients (1988‐2013) were included in our study, of whom 668 patients underwent surgery alone and 609 underwent surgery +RT (Table 1). Significant differences were found with respect to the variables of age (*P* < 0.001), year of diagnosis (*P* < 0.001), tumor sites (*P* < 0.001), histologic types (*P* < 0.001), tumor size (*P* = 0.001), and surgery type (*P* < 0.001). The median follow‐up time was 77 months for the surgery alone group and was 63 months for surgery +RT group, which had no significant differences. Table 1 shows the baseline characteristics of patients and tumors. Elderly, astrocytoma histology or no gross total resection patients seemed more likely to receive PORT by surgeons. There were no significant differences in sex and race/ethnicity between the two groups.

**Table 1:** Baseline characteristics of postoperative patients with adult low‐grade(WHO grade II) infiltrative supratentorial astrocytoma/oligodendroglioma

Characteristic	No. (%) of patients			*P*
	Total (n = 1277)	Surgery alone (n = 668)	Surgery +RT (n = 609)	
Age, y, mean ±SD	41.3 ± 14.2	38.8 ± 14.0	44 ± 13.9	<0.001
Age group, y				<0.001
≤40	708 (55.4)	419 (62.7)	289 (47.5)	
>40	569 (44.6)	249 (37.3)	320 (52.5)	
Sex				0.369
Male	772 (60.5)	396 (59.3)	376 (61.7)	
Female	505(39.5)	272 (40.7)	233 (38.3)	
Race/ethnicity				0.271
White	1133 (88.7)	585 (87.6)	548 (90.0)	
Black	67(5.2)	36 (5.4)	31 (5.1)	
Others	77 (6.0)	47 (7.0)	30 (4.9)	
Year of diagnosis				<0.001
1988‐1995	243 (19.0)	73 (10.9)	170 (27.9)	
1996‐2004	543 (42.5)	273 (40.9)	270 (44.3)	
2005‐2013	491 (38.4)	322 (48.2)	169 (27.8)	
Tumor sites				<0.001
Frontal lobe	636 (49.8)	370 (55.4)	266 (43.7)	
Temporal lobe	248 (19.4)	132 (19.8)	116 (19.0)	
Parietal lobe	150 (11.7)	66 (9.9)	84 (13.8)	
Occipital lobe	23 (1.8)	13 (1.9)	10 (1.6)	
Others	220 (17.2)	87 (13.0)	133 (21.8)	
Histologic types				<0.001
Astrocytoma	696 (54.5)	323 (48.4)	373 (61.2)	
Oligodendroglioma	581 (45.5)	345 (51.6)	236 (38.8)	
Tumor size, mm, mean ±SD	43.6 ± 20.5	41.7 ± 20.9	45.6 ± 19.9	0.001
Surgical type				<0.001
Gross total resection	515 (40.3)	333 (49.9)	182 (29.9)	
Subtotal resection	431 (33.8)	171 (25.6)	260 (42.7)	
Biopsy	331 (25.9)	164 (24.6)	167 (27.4)	
Median(range)follow‐up time, mo	69 (0‐306)	77 (0‐290)	63 (0‐306)	0.156

RT, radiotherapy.

**Figure 1:**
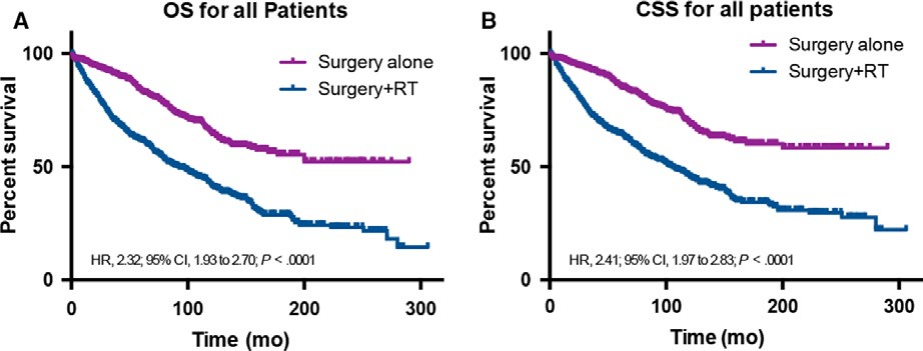
Overall (A) and cancer–specific (B) survivals in all patients with adult low‐grade (WHO grade II) infiltrative supratentorial astrocytoma/oligodendroglioma (ALISA/O) undergoing surgery alone or surgery +RT. CSS, cancer‐specific survival; HR, hazard ratio; OS, overall survival; RT, radiotherapy

According to survival analysis by log‐rank test, surgery +RT rather than surgery alone was significantly associated with worse OS (hazard ratio [HR], 2.32; 95% CI, 1.93‐2.70; *P* < 0.001) and CSS (HR, 2.41; 95% CI, 1.97‐2.83; *P* < 0.001) for patients with ALISA/O (Figure 1A,B).

### 3.2 The optimal cutoff value for tumor size calculated with X‐tile

**Figure 2:**
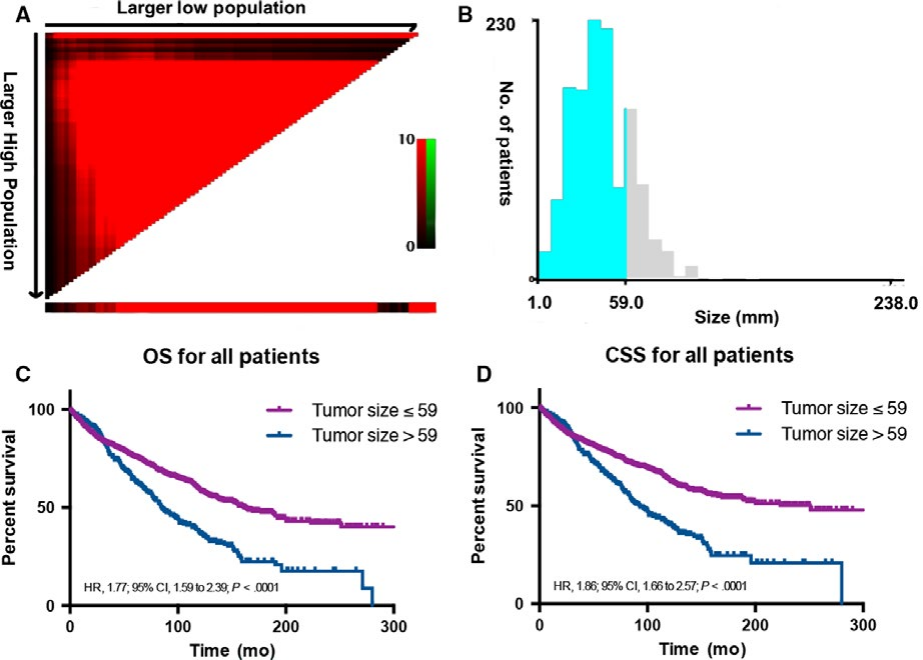
X‐tile analysis of survival data from the SEER registry reveals a continuous distribution based on tumor size, equally divided into training and validation sets. A shows tumor size divided at the optimal cut‐point, as defined by the most significant (brightest pixel) on the plot (59 mm, *P* < 0.0001). Diffuse red indicates a continuous indirect association between increasing tumor size and good prognosis. B shows the cut‐point on a histogram of the entire cohort. Kaplan‐Meier analysis is provided to analyze overall (C) and cancer–specific (D) survivals based on optimal tumor size cutoff point in the whole cohort. *P* values were determined using the cutoff point defined in the training set and applying it to the validation set. (The optimal cutoff value for tumor size is 59 mm, χ2 = 50.107, *P* < 0.001). CSS, cancer‐specific survival; HR, hazard ratio; OS, overall survival.

Individual results were analyzed using different tumor sizes ranging from 10 to 80 mm to assess the influence of tumor size on CSS. The CSS was calculated for patients with larger or smaller than the tumor size. As shown in Table 2, tumor size in postoperative patients with ALISA/O was a prognosis factor for size ranging from 10 to 80 mm. With the tumor size increasing from 10 to 80 mm, the CSS rate increased from 34.4% to 76.1% (Table 2). Next, X‐tile plots^13^ were constructed, and the maximum chi‐square log‐rank value of 50.107 (the tumor size as 59 mm, Figure 2A,B, *P* < 0.001) was produced, identifying 59 mm as the optimal cutoff point to divide the cohort into high‐ and low risk groups in terms of CSS. A significant improvement in CSS was observed between two groups (67.4% vs 44.4%, Table 2). The K‐M analysis also showed significant difference in OS (HR, 1.77; 95% CI, 1.59‐2.39; *P*0< 0.001) and CSS (HR, 1.86; 95% CI, 1.66‐2.57; *P* < 0.001) between two subsets (Figure 2C,D).

**Table 2:** Univariate analysis of the influence of different tumor size for CSS in all patients who received surgery (n = 1277)

Tumor size （mm）	No. of patients	CSS	RR	χ2	*P*
≤10	46	76.1%	ref	4.760	0.342
>10	1231	61.3%	1.62		
≤20	185	74.1%	ref	14.127	0.006
>20	1092	59.7%	1.55		
≤30	400	72.3%	ref	23.389	0.000
>30	877	57.0%	1.55		
≤40	658	67.9%	ref	20.269	0.001
>40	619	55.3%	1.40		
≤50	884	66.5%	ref	29.870	0.000
>50	393	51.2%	1.46		
≤59	966	67.4%	ref	50.107	0.000
>59	311	44.4%	1.71		
≤60	1068	65.1%	ref	33.407	0.000
>60	209	45.0%	1.58		
≤70	1181	64.0%	ref	45.946	0.000
>70	96	34.4%	1.82		
≤80	1234	62.6%	ref	15.796	0.003
>80	43	37.2%	1.68		

CSS, cancer‐specific survival.

### 3.3 The effects of tumor size and port for postoperative patients in low‐ and high risk groups before and after PSM

Then, we divided 1277 patients into low‐ (n = 323) and high risk (n = 954) groups. In the low risk group, 229 patients were receiving surgery alone and 94 patients receiving surgery +RT, while 439 patients receiving surgery alone and 515 patients were receiving surgery +RT in the high risk group. Significant differences emerged in year of diagnosis (*P* < 0.001) in the low risk group and year of diagnosis (*P* < 0.001), tumor sites (*P* < 0.001), histologic types (*P* < 0.001), surgery type (*P* < 0.001) in the high risk group. Therefore, PSM was performed to balance the above variables. After PSM, 87 patients in the surgery +RT group were matched to 87 patients in the surgery alone group for low‐risk patients, while 247 patients in the surgery +RT group were matched to 247 patients in the surgery alone group for high risk patients. There was no significance in any variable within the group of low risk or high risk (Tables 3 and 4).

**Table 3:** Baseline characteristics of patients in low‐ and high risk before PSM

Characteristic	No.(%) of patients					
	Low risk(n = 323)			High risk(n = 954)		
	Surgery alone (n = 229)	Surgery +RT (n = 94)	*P*	Surgery alone (n = 439)	Surgery +RT (n = 515)	*P*
Age group, years			/			0.089
≤40	229(100)	94(100)		190(43.3)	195(37.9)	
>40	0(0)	0(0)		249(56.7)	320(62.1)	
Sex			0.598			0.117
Male	146(63.8)	57(60.6)		250(56.9)	319(61.9)	
Female	83(36.2)	37(39.4)		189(43.1)	196(38.1)	
Race/ethnicity			0.911			0.286
White	198(86.5)	82(87.2)		387(88.2)	466(90.5)	
Black	16(7.0)	7(7.4)		20(4.6)	24(4.7)	
Others	15(6.6)	5(5.3)		32(7.3)	25(4.9)	
Year of diagnosis			<0.001			<0.001
1988‐1995	17(7.4)	23(24.5)		56(12.8)	147(28.5)	
1996‐2004	116(50.7)	42(44.7)		157(35.8)	228(44.3)	
2005‐2013	96(41.9)	29(30.9)		226(51.5)	140(27.2)	
Tumor sites			0.759			<0.001
Frontal lobe	129(56.3)	56(59.6)		241(54.9)	210(40.8)	
Temporal lobe	51(22.3)	17(18.1)		81(18.5)	99(19.2)	
Parietal lobe	15(6.6)	9(9.6)		51(11.6)	75(14.6)	
Occipital lobe	4(1.7)	2(2.1)		9(2.1)	8(1.6)	
Others	30(13.1)	10(10.6)		57(13.0)	123(23.9)	
Histologic types			0.222			<0.001
Astrocytoma	112(48.9)	53(56.4)		211(48.1)	320(62.1)	
Oligodendroglioma	117(51.1)	41(43.6)		228(51.9)	195(37.9)	
Tumor size group, mm			0.078			0.194
≤59	188(82.1)	69(73.4)		335(76.3)	374(72.6)	
>59	41(17.9)	25(26.6)		104(23.7)	141(27.4)	
Surgical type			/			0.001
Gross total resection	229(100)	94(100)		104(23.7)	88(17.1)	
Subtotal resection	0(0)	0(0)		171(39.0)	260(50.5)	
Biopsy	0(0)	0(0)		164(37.4)	167(32.4)	
Median(range)follow‐up time, months	95(0‐258)	83(5‐263)	0.964	64(0‐290)	57(0‐306)	0.850

PSM, propensity score match; RT, radiotherapy.

**Table 4:** Baseline Characteristics of Patients in Low‐ and High‐Risk After PSM

Characteristic	No.(%) of Patients					
	Low risk(n = 174)			High risk(n = 494)		
	Surgery alone (n = 87)	Surgery +RT (n = 87)	*P*	Surgery alone (n = 247)	Surgery +RT (n = 247)	*P*
Age group, years			/			0.928
≤40	87(100)	87(100)		106(42.9)	105(42.5)	
>40	0(0)	0(0)		141(57.1)	142(57.5)	
Sex			0.527			1.000
Male	58(66.7)	54(62.1)		146(59.1)	146(59.1)	
Female	29(33.3)	33(37.9)		101(40.9)	101(40.9)	
Race/ethnicity			0.806			0.716
White	76(87.4)	75(86.2)		229(92.7)	224(90.7)	
Black	5(5.7)	7(8.0)		8(3.2)	10(4.0)	
Others	6(6.9)	5(5.7)		10(4.0)	13(5.3)	
Year of diagnosis			0.303			0.813
1988‐1995	13(14.9)	18(20.7)		41(16.6)	38(15.4)	
1996‐2004	50(57.5)	40(46.0)		110(44.5)	117(47.4)	
2005‐2013	24(27.6)	29(33.3)		96(38.9)	92(37.2)	
Tumor sites			0.801			0.774
Frontal lobe	54(62.1)	51(58.6)		131(53.0)	140(56.7)	
Temporal lobe	17(19.5)	16(18.4)		50(20.2)	43(17.4)	
Parietal lobe	5(5.7)	9(10.3)		24(9.7)	27(10.9)	
Occipital lobe	1(1.1)	2(2.3)		4(1.6)	2(0.8)	
Others	10(11.5)	9(10.3)		38(15.4)	35(14.2)	
Histologic types			0.095			0.588
Astrocytoma	38(43.7)	49(56.3)		130(52.6)	136(55.1)	
Oligodendroglioma	49(56.3)	38(43.7)		117(47.4)	111(44.9)	
Tumor size group, mm			0.603			0.459
≤59	63(72.4)	66(75.9)		192(77.7)	185(74.9)	
>59	24(27.6)	21(24.1)		55(22.3)	62(25.1)	
Surgical type			/			0.923
Gross total resection	87(100)	87(100)		56(22.7)	54(21.9)	
Subtotal resection	0(0)	0(0)		118(47.8)	116(47.0)	
Biopsy	0(0)	0(0)		73(29.6)	77(31.2)	
Median(range)follow‐up time, months	106(0‐258)	82(5‐259)	0.047	79(0‐290)	67(0‐285)	0.415

PSM, propensity score match; RT, radiotherapy.

**Figure 3:**
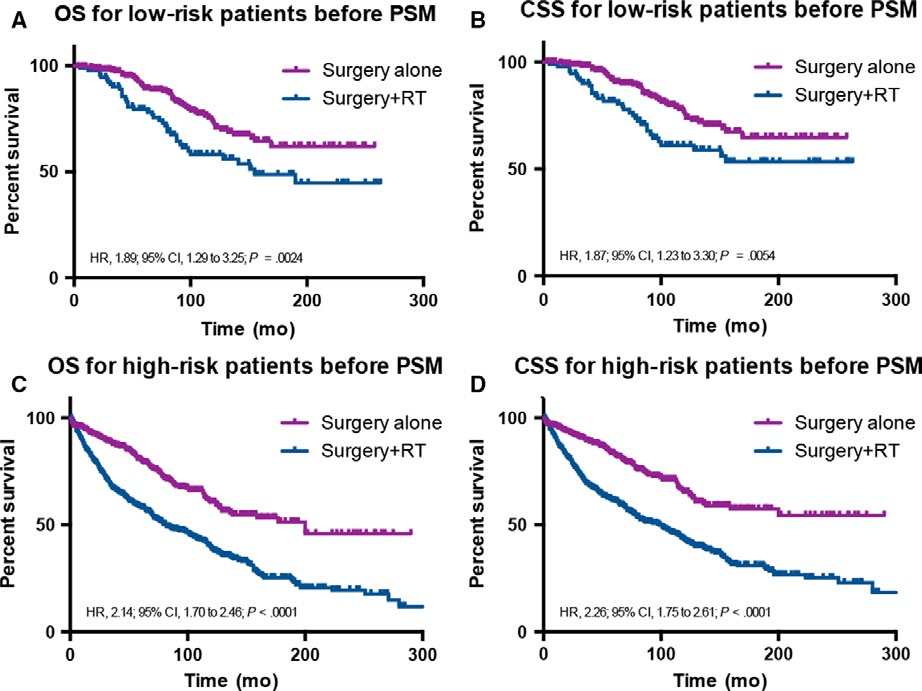
Kaplan‐Meier survival curves illustrating ALISA/O overall and cancer‐specific survival between the surgery alone and surgery +RT groups for low‐ (A and B) and high‐risk (C and D) patients before propensity score matching. CSS, cancer‐specific survival; HR, hazard ratio; OS, overall survival; PSM, propensity score matched; RT, radiotherapy.

Before PSM, we analyzed OS and CSS of the surgery alone vs surgery +RT groups using the Kaplan‐Meier analysis and log‐rank test (Figure 3). In the low risk group, the survival analysis showed that surgery +RT rather than surgery alone was significantly associated with worse OS (HR, 1.89; 95% CI, 1.29‐3.25; *P* = 0.0024) and CSS (HR, 1.87; 95% CI, 1.23‐3.30; *P* = 0.0054; Figure 3A,B). A similar trend was found in the high risk group, with worse OS (HR, 2.14; 95% CI, 1.70‐2.46; *P* < 0.001) and CSS (HR, 2.26; 95% CI, 1.75‐2.61; *P* < 0.001; Figure 3C,D). Using the surgery alone group as the reference, multivariable analysis was performed to determine the HR (95% CI) of OS (HR, 1.51; 95% CI, 0.96‐2.39; *P* = 0.077) and CSS (HR, 1.51; 95% CI, 0.93‐2.46; *P* = 0.100) in the low risk group (Table 5). In the high risk group, there were significant differences with worse OS (HR, 1.46; 95% CI, 1.18‐1.79; *P* < 0.001) and CSS (HR, 1.48; 95% CI, 1.18‐1.85; *P* < 0.001) using the same reference in multivariable analysis (Table 5). What is more, using tumor size ≤59 mm as the reference, the HR of OS was 3.907 (95% CI, 2.275‐6.709, *P* < 0.001), and the HR of CSS was 3.575 (95% CI, 2.031‐6.293, *P* < 0.001). In the high risk group, the HR of OS was 1.689 (95% CI, 1.243‐2.295, *P* = 0.001), and the HR of CSS was 1.852 (95% CI, 1.338‐2.562, *P* < 0.001; Table 5). Moreover, the results also revealed that age, year of diagnosis, tumor sites, histologic types, and surgery type were independent prognostic factors for CSS in the high risk group.

**Table 5:** Cox proportional hazards regression model for overall survival and cancer‐specific survival in low‐ and high‐risk patients before PSM

Variable	Low risk (n = 323)				High risk (n = 954)			
	OS		CSS		OS		CSS	
	Hazard ratio (95% CI)	*P*	Hazard ratio (95% CI)	*P*	Hazard ratio (95% CI)	*P*	Hazard ratio (95% CI)	*P*
Age group, y								
≤40	1.00 (reference)		1.00 (reference)		1.00 (reference)		1.00 (reference)	
>40	—	—	—	—	2.072 (1.687‐2.545)	0.000	1.848 (1.490‐2.291)	0.000
Sex								
Male	1.00 (reference)		1.00 (reference)		1.00 (reference)		1.00 (reference)	
Female	0.929 (0.602‐1.435)	0.740	1.008 (0.639‐1.590)	0.973	0.918 (0.760‐1.110)	0.378	0.860 (0.701‐1.055)	0.149
Race/ethnicity		0.325		0.664		0.113		0.290
White	1.00 (reference)		1.00 (reference)		1.00 (reference)		1.00 (reference)	
Black	1.646 (0.775‐3.497)	0.195	1.395 (0.591‐3.293)	0.447	1.524 (1.019‐2.278)	0.040	1.420 (0.918‐2.197)	0.115
Others	0.735 (0.294‐1.839)	0.511	0.815 (0.325‐2.046)	0.664	1.116 (0.747‐1.666)	0.593	1.028 (0.660‐1.600)	0.904
Year of diagnosis		0.052		0.050		0.001		0.002
1988‐1995	1.00 (reference)		1.00 (reference)		1.00 (reference)		1.00 (reference)	
1996‐2004	1.157 (0.655‐2.043)	0.616	1.139 (0.623‐2.082)	0.672	0.759 (0.606‐0.951)	0.016	0.775 (0.610‐0.985)	0.037
2005‐2013	0.507 (0.226‐1.139)	0.100	0.458 (0.190‐1.103)	0.082	0.592 (0.446‐0.787)	0.000	0.581 (0.429‐0.786)	0.000
Tumor sites		0.593		0.635		0.009		0.001
Frontal lobe	1.00 (reference)		1.00 (reference)		1.00 (reference)		1.00 (reference)	
Temporal lobe	0.635 (0.356‐1.134)	0.125	0.681 (0.372‐1.245)	0.212	1.252 (0.969‐1.618)	0.086	1.314 (0.997‐1.732)	0.053
Parietal lobe	0.860 (0.336‐2.201)	0.754	0.581 (0.177‐1.902)	0.369	1.413 (1.055‐1.892)	0.020	1.536 (1.126‐2.097)	0.007
Occipital lobe	0.570 (0.076‐4.265)	0.584	0.695 (0.092‐5.249)	0.725	1.500 (0.761‐2.956)	0.241	1.587 (0.772‐3.262)	0.209
Others	1.051 (0.562‐1.966)	0.876	1.098 (0.572‐2.108)	0.779	1.515 (1.189‐1.931)	0.001	1.718 (1.329‐2.222)	0.000
Histologic types								
Astrocytoma	1.00 (reference)		1.00 (reference)		1.00 (reference)		1.00 (reference)	
Oligodendroglioma	0.664 (0.434‐1.016)	0.059	0.740 (0.471‐1.162)	0.190	0.502 (0.409‐0.617)	0.000	0.436 (0.347‐0.546)	0.000
Tumor size group, mm								
≤59	1.00 (reference)		1.00 (reference)		1.00 (reference)		1.00 (reference)	
>59	4.392 (2.825‐6.827)	0.000	4.323 (2.716‐6.883)	0.000	1.504 (1.230‐1.838)	0.000	1.561 (1.261‐1.933)	0.000
Surgical type		—		—		0.00		0.000
Gross total resection	1.00 (reference)		1.00 (reference)		1.00 (reference)		1.00 (reference)	
Subtotal resection	—	—	—	—	1.673 (1.253‐2.234)	0.000	1.908 (1.373‐2.652)	0.000
Biopsy	—	—	—	—	1.936 (1.438‐2.606)	0.000	2.221 (1.585‐3.110)	0.000
PORT								
No	1.00 (reference)		1.00 (reference)		1.00 (reference)		1.00 (reference)	
Yes	1.511 (0.956‐2.388)	0.077	1.508 (0.924‐2.461)	0.100	1.456 (1.184‐1.789)	0.000	1.477 (1.182‐1.845)	0.001

CSS, cancer‐specific survival; PORT, postoperative radiotherapy; PSM, propensity score match; OS, overall survival.

**Figure 4:**
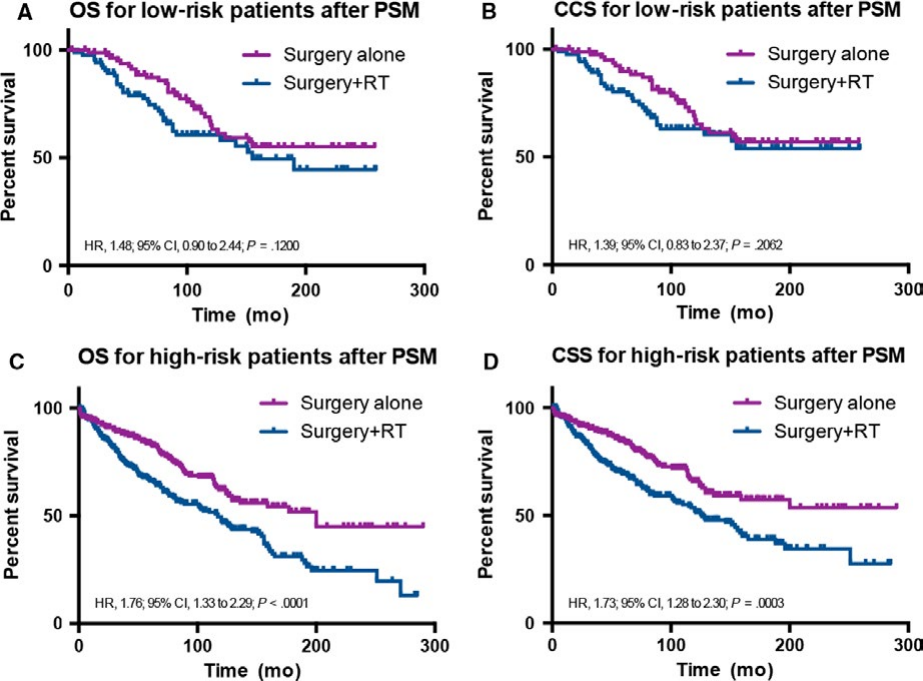
Kaplan‐Meier survival curves illustrating ALISA/O overall and cancer‐specific survival between the surgery alone and surgery +RT groups for low‐ (A and B) and high‐risk (C and D) patients after propensity score matching. CSS, cancer‐specific survival; HR, hazard ratio; OS, overall survival; PSM, propensity score matched; RT, radiotherapy

After PSM, unadjusted OS and CSS of the surgery alone vs surgery +RT group for low‐ and high risk patients, using the Kaplan‐Meier method and log‐rank test, were also determined. In patients with high‐risk, significant degradations of OS and CSS were observed between surgery alone and surgery +RT in OS (surgery +RT vs surgery alone: HR, 1.76; 95% CI, 1.33‐2.29; *P* < 0.001) and in CSS (surgery +RT vs surgery alone: HR, 1.73; 95% CI, 1.28‐2.30; *P* < 0.001; Figure 4A,B). In the group with low risk patients, there was no significant difference in survival between surgery +RT and surgery alone (OS: HR, 1.48; 95% CI, 0.90‐2.44; *P* = 0.120; CSS: HR, 1.39; 95% CI, 0.83‐2.37; *P* = 0.206; Figure 4C,D). Using the surgery alone group as the reference in multivariable analysis, we found that the HR of OS was 1.535 (95% CI, 0.916‐2.574; *P* = 0.104), the HR of CSS was 1.482 (95% CI, 0.863‐2.546; *P* = 0.154) in the low risk group and the HR of OS was 1.482 (95% CI, 1.127‐1.950; *P* = 0.005), the HR of CSS was 1.440 (95% CI, 1.073‐1.933; *P* = 0.015) in the high risk group (Table 6), which maintained consistency with the analysis before PSM. Comparing tumor size >59 mm group with tumor size ≤59 mm, multivariable analysis showed tumor size >59 mm was independently associated with diminished OS and CSS both in the low‐ and high risk groups. (OS in the low risk group: HR, 3.907; 95% CI, 2.275‐6.709; *P* < 0.001; CSS in the low risk group: HR, 3.575; 95% CI, 2.031‐6.293; *P* < 0.001; OS in the high risk group: HR, 1.689; 95% CI, 1.243‐2,295; *P* = 0.001; CSS in the high risk group: HR, 1.852; 95% CI, 1.338‐2.562; *P* < 0.001; Table 6). Other independent prognostic factors for CSS in the high risk group were age, tumor sites, histologic types, and surgery type after PSM, which eliminated year of diagnosis comparing with the data before PSM.

**Table 6:** Cox proportional hazards regression model for overall survival and cancer‐specific survival in low‐ and high‐risk patients after PSM

Variable	Low risk (n = 174)				High risk (n = 494)			
	OS		CSS		OS		CSS	
	Hazard ratio (95% CI)	*P*	Hazard ratio (95% CI)	*P*	Hazard ratio (95% CI)	*P*	Hazard ratio (95% CI)	*P*
Age group, y								
≤40	1.00 (reference)		1.00 (reference)		1.00 (reference)		1.00 (reference)	
>40	—	—	—	—	1.868 (1.378‐2.532)	0.000	1.665 (1.206‐2.298)	0.002
Sex								
Male	1.00 (reference)		1.00 (reference)		1.00 (reference)		1.00 (reference)	
Female	0.859 (0.504‐1.464)	0.576	0.864 (0.494‐1.512)	0.609	0.936 (0.706‐1.242)	0.648	0.934 (0.689‐1.266)	0.661
Race/ethnicity		0.507		0.455		0.101		0.227
White	1.00 (reference)		1.00 (reference)		1.00 (reference)		1.00 (reference)	
Black	1.503 (0.622‐3.631)	0.365	1.641 (0.675‐3.987)	0.274	1.714 (0.920‐3.195)	0.090	1.740 (0.901‐3.359)	0.099
Others	0.672 (0.207‐2.183)	0.509	0.729 (0.224‐2.375)	0.600	1.522 (0.872‐2.657)	0.140	1.244 (0.657‐2.358)	0.503
Year of diagnosis		0.178		0.157		0.463		0.610
1988‐1995	1.00 (reference)		1.00 (reference)		1.00 (reference)		1.00 (reference)	
1996‐2004	1.149 (0.595‐2.217)	0.679	1.135 (0.574‐2.242)	0.716	0.965 (0.653‐1.425)	0.856	0.968 (0.636‐1.474)	0.881
2005‐2013	0.495 (0.179‐1.364)	0.174	0.439 (0.148‐1.299)	0.137	0.780 (0.496‐1.227)	0.282	0.812 (0.506‐1.303)	0.388
Tumor sites		0.946		0.925		0.091		0.030
Frontal lobe	1.00 (reference)		1.00 (reference)		1.00 (reference)		1.00 (reference)	
Temporal lobe	0.826 (0.408‐1.674)	0.596	0.979 (0.477‐2.009)	0.955	1.473 (1.019‐2.131)	0.039	1.558 (1.052‐2.308)	0.027
Parietal lobe	1.161 (0.440‐3.064)	0.763	0.772 (0.230‐2.592)	0.675	1.609 (1.010‐2.561)	0.045	1.773 (1.083‐2.900)	0.023
Occipital lobe	0.888 (0.112‐7.069)	0.911	1.081 (0.135‐8.686)	0.941	1.798 (0.534‐6.049)	0.343	2.460 (0.722‐8.384)	0.150
Others	1.203 (0.562‐2.579)	0.634	1.362 (0.630‐2.944)	0.432	1.425 (0.966‐2.100)	0.074	1.588 (1.050‐2.401)	0.028
Histologic types								
Astrocytoma	1.00 (reference)		1.00 (reference)		1.00 (reference)		1.00 (reference)	
Oligodendroglioma	0.809 (0.478‐1.370)	0.431	0.897 (0.515‐1.562)	0.702	0.529 (0.393‐0.712)	0.000	0.474 (0.342‐0.657)	0.000
Tumor size group, mm								
≤59	1.00 (reference)		1.00 (reference)		1.00 (reference)		1.00 (reference)	
>59	3.907 (2.275‐6.709)	0.000	3.575 (2.031‐6.293)	0.000	1.689 (1.243‐2.295)	0.001	1.852 (1.338‐2.562)	0.000
Surgical type		—		—		0.069		0.026
Gross total resection	1.00 (reference)		1.00 (reference)		1.00 (reference)		1.00 (reference)	
Subtotal resection	—	—	—	—	1.597 (1.053‐2.422)	0.027	1.883 (1.165‐3.043)	0.010
Biopsy	—	—	—	—	1.586 (1.023‐2.458)	0.039	1.879 (1.138‐3.102)	0.014
PORT								
No	1.00 (reference)		1.00 (reference)		1.00 (reference)		1.00 (reference)	
Yes	1.535 (0.916‐2.574)	0.104	1.482 (0.863‐2.546)	0.154	1.482 (1.127‐1.950)	0.005	1.440 (1.073‐1.933)	0.015

CSS, cancer‐specific survival; PORT, postoperative radiotherapy; PSM, propensity score match; OS, overall survival.

### 3.4 Prognostic nomogram for CSS in the high risk group after PSM

**Figure 5:**
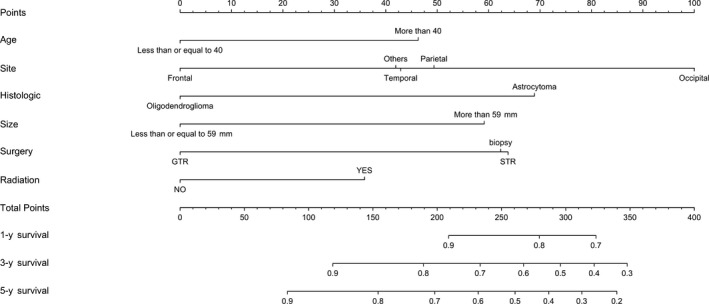
A cancer‐specific survival nomogram for high risk patients with ALISA/O. To use the nomogram, an individual patient's value is located on each variable axis, and a line is drawn upward to determine the number of points received for each variable value. The sum of these numbers is located on the Total Points axis, and a line is drawn downward to the survival axes to determine the likelihood of 1‐, 3‐, or 5‐year survival

**Figure 6:**
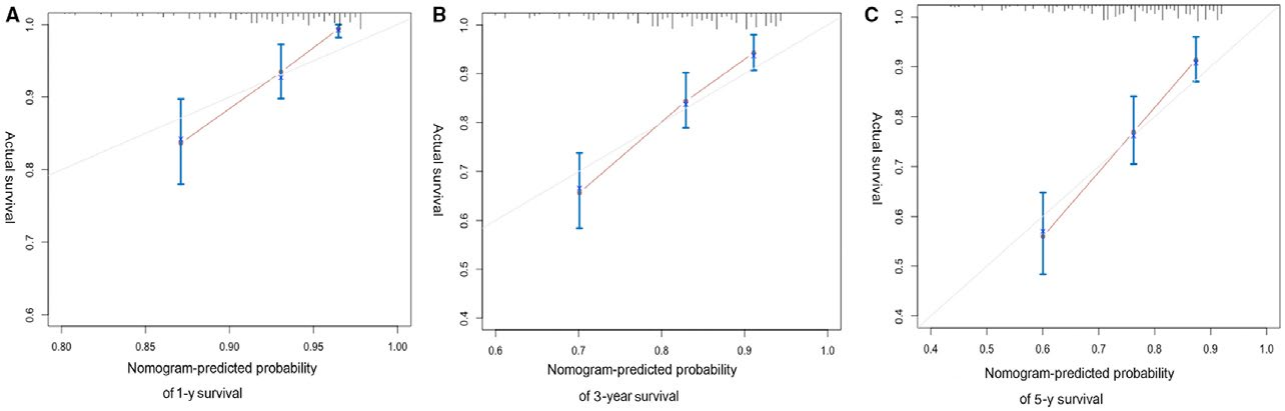
The calibration curve for predicting patient survival at (A) one year, (B) three year, and (C) five year in the primary cohort. Nomogram‐predicted probability of cancer‐specific survival is plotted on the *x*‐axis; actual cancer‐specific survival is plotted on the *y*‐axis

On multivariate analysis of the primary cohort in the high‐risk group after PSM, independent factors for CSS were age group, tumor sites, histologic types, size group, surgery type, and PORT, which were all selected into the nomogram (Figure 5). The C‐index of the nomogram was 0.68 (95% CI, 0.65‐0.72) according to predicting survival. The survival probability calibration curve (Figure 6) was in good agreement between prediction by nomogram and actual observation.

## 4 DISCUSSION

The guidelines had recommended tumor size as an indicator to distinguish between low‐ and high‐risk for postoperative patients with ALISA/O. Inadequate tumor size grading would affect the next treatment. Therefore, it is of great significance to develop effective tumor size classification based on survival to guide further treatment. Conventional methods can be used to group tumor size by referring to references, guidelines, or consensus. But, this method was subjective and may reduce the chance of detecting differences.

Several studies, investigating the prognostic factors of ALISA/O, had found tumor size was a significant predictor for survival.^5^, ^6^, ^7^, ^8^, ^9^ They have tried to evaluate the survival difference in tumor size for ALISA/O patients. Authoritatively, conducting a multivariate analysis of two high‐quality EORTC phase III trials, Pignatti et al^5^ revealed that a tumor size ≥6 cm was an unfavorable factor for patients with cerebral low‐grade glioma, which was widely accepted. Based on a large population, we obtained very similar results. We used X‐tile^13^ to identify the optimal cutoff point for tumor size as 59 mm with the maximum chi‐squared value. The K‐M analysis showed that tumor size >59 mm had significantly worse OS and CSS. Both before and after PSM, multivariable Cox proportional hazard regression models showed tumor size >59 mm was independently associated with diminished OS and CSS in the low‐ and high risk groups. Thus, these findings strongly confirm 59 mm was a reasonable tumor size cutoff point for patients with ALISA/O.

Management of patients with ALISA/O (WHO grade II) has mainly concentrated in prognostic factors. One issue is its relatively long life span compared to other CNS tumors, which makes it important to take benefits and potential damage of treatment such as surgery, radiation, and chemotherapy into consideration. Early and maximal surgical resection was now fully recognized as the first therapeutic option whenever feasible.^16^ At a time when neither modern imaging technology nor alternative treatment modalities were available, radiation therapy (RT) was the accepted treatment for progressive and inoperable low‐grade glioma. Several studies concluded that patients with low‐grade glioma demonstrated a tumor volume decrease,^17^ improved seizure outcome,^18^ and prolonged both progressive‐free survival (PFS) and overall survival^19^ after RT. In addition, other studies suggested that PORT was associated with improved PFS and was recommended for patients receiving subtotal resection or biopsy only.^8^, ^20^, ^21^ However, because of the low level of evidence in these reports, the influence of PORT still should be verified in future phase III trials. The debate still continues about whether PORT could be a beneficial treatment for ALISA/O patients.

As aforementioned studies have found, it is reasonable to assume that PORT could be adequate for ALISA/O patients. However, evidence from our study conflicts with that assumption that ALISA/O was resistant to PORT. Our results showed that surgery alone was superior to surgery +RT for ALISA/O patients both in the low‐ and high risk groups. There was no significant difference for low risk patients. We found selective biases between the surgery alone and surgery +RT groups, resulting in failure to draw an accurate conclusion. We used an advanced statistical method, named propensity score matched (PSM) to control for the biases of covariates, and we obtained the same result. The most likely reason for no significant differences in the low risk group was the weaker power of sample size in this group, which had approximately 65% less samples than the high risk group before or after PSM. Therefore, because of a larger sample size in the high risk group, a difference in survival could be observed in patients with ALISA/O who underwent surgery alone vs surgery +RT. Furthermore, we believe that high‐quality evidence from future randomized controlled trials is needed to verify our results.

The reasons why patients receiving RT had a worse prognosis deserve further discussion. Several studies found that radiotherapy has been associated with additional long‐term cognitive disability^22^, ^23^, ^24^ and impaired executive function,^25^ as well as potential fatal radiation necrosis even at low doses.^7^ Toward the end of the last century, a randomized controlled trial by Kiebert et al^26^ provided evidence for high dose RT reported significantly decreased levels of functioning and a higher symptom burden for postoperative patients. Although data suggesting that radiation is relatively safe when lower fractions are used,^27^ patients and physicians were often hesitant to proceed with this treatment. The use of PORT for patients with ALISA/O has declined in the period from 1998 to 2006 for both low‐ and high risk patients.^28^ Recent studies indicate a role in the treatment of ALISA/O for chemotherapy, which could offer an alternative to radiotherapy.^29^ What is more, a treatment strategy combining radiation and chemotherapy has been confirmed with a clear advantage for ALISA/O patients.^30^, ^31^

Considering the adverse effects of PORT, a strategy of “wait and see” with strict observation on MRI until disease progression has been advocated in patients with ALISA/O who have had an extensive resection. A prospective study of observation in low risk patients with ALISA/O found that slightly more than 50% recurred within 5 years of surgery.^10^ Thus, strict observation is a reasonable option for postoperative low risk patients. Observation is sometimes elected for high risk patients who have had surgery, but the risk is correspondingly increased.

Following the recommendations in the guideline and the results of our study, age, surgery type, and tumor size have been identified as important prognostic factors. But the role of tumor sites, histologic types and PORT should also be taken into account to determine prognosis for high risk patients with ALISA/O. These factors were used to derive a prognostic scoring system, nomogram, that can be readily calculated based on the total scores of prognostic factors present, with increasing scores corresponding to worse survival.

We have to admit that there are some limitations in our study. First, although we have used PSM to balance the obvious significant covariates between groups, some potential biases are still difficult to avoid because this was a retrospective study. We hope that there will be more evidence‐based research in the future to confirm our results. Second, we cannot obtain detail information of PORT, such as radiation dose, field, and duration, limiting our further research because of the inherent defects in the SEER database. Also, the SEER database cannot provide the information of postoperative chemotherapy. Yet, patients with ALISA/O often receive this therapy; thus, this limitation confounds our results and makes it difficult to assess the role of PORT accurately. As a final comment, the SEER database lacks the special code for pilomyxoid astrocytoma, which affected the integrity of our study. We recommend that the SEER database can incorporate the code of this tumor subtype in the future.

## 5 CONCLUSION

Comparing with 2017 NCCN guideline (CNS cancer), we undertook this study to assess two changes of its update in 2018 for patients with ALISA/O in a population‐based cohort. We concluded that tumor size of 59 mm was an optimal cutoff point to divide patients into relatively low‐ or high risk subgroups and that PORT may not benefit patients. The nomogram established in our study objectively and accurately predicted the prognosis for high risk patients with ALISA/O. Additional studies are required to verify our conclusion.

## CONFLICT OF INTEREST

None declared.
